# Achieving sustainable development nutrition targets: the challenge for South Asia

**DOI:** 10.7189/jogh.10.010303

**Published:** 2020-06

**Authors:** Deepali Sharma

**Affiliations:** 1 Tata Institute of Social Sciences, Mumbai, India; 2 SGTB Khalsa College, University of Delhi, India

The high levels of malnutrition prevailing worldwide are a cause for concern, despite the importance accorded to addressing the problem in the Sustainable Development Goals (SDG) 2030 Agenda. The situation is even more alarming in South Asia with the region having the highest numbers of malnourished children. It is extremely important that the region meets the nutrition and health goals, if the global goals are to be achieved. The aim is to highlight the levels and trends in malnutrition within South Asia and project the progress towards the SDGs. While the region is lagging behind in most of the nutrition indicators, it is found that that the region has very high rates of anemia for women in the reproductive age group. South Asia would miss the targets unless urgent action is taken. This study reiterates the well-established fact that the role of women needs to be strengthened within the region.

High levels of malnutrition continue to prevail worldwide despite the importance accorded to the issue in the Sustainable Development Goals (SDG) 2030 Agenda. The SDGs hold, correctly, that nutrition has a multidirectional relationship with many developmental goals, and thus, it is of vital importance that countries achieve Target 2.2 of the SDGs: “by 2030 end all forms of malnutrition, including achieving by 2025 the internationally agreed targets on stunting and wasting in children under five years of age, and address the nutritional needs of adolescent girls, pregnant and lactating women, and older persons”. This target is based on the goals outlined by the 65^th^ World Health Assembly (WHA) in May 2012 for fighting all forms of malnutrition globally. The WHA emphasises five key indicators that reveal the status of nutrition in nations: prevalence in children under five of stunting (low height-for-age), wasting (low weight-for-height) and overweight (high weight-for-height); the percentage of infants younger than six months who are exclusively breastfed; and the percentage of reproductive-age women (15-49 years) with anaemia.

Globally, curbing malnutrition remains a mammoth challenge. While stunting is declining globally among children under five years of age, wasting is on the rise. In parallel, the phenomenon of overnutrition – or the rise of overweight and obesity – is on the rise in almost every country. To compound the problem, a large proportion of the population is affected by micronutrient deficiencies, or ‘hidden hunger’. This is the triple burden of malnutrition afflicting most of the countries today. In our opinion one of the greatest, yet not discussed as much, concerns is anaemia, especially anaemia among women – anaemia in women impairs not only their own health and well-being, but it also increases the risk of adverse maternal and neonatal outcomes and perpetuates a cross-generational cycle of malnutrition.

In the following sections, we examine the levels and trends in nutrition indicators in the South Asian countries. We also present projection results for three countries – India, Nepal, and Bangladesh – for the five WHA nutrition indicators and their likelihood of achieving the SDG by 2030. It is seen that despite rapid economic development in the region, malnutrition continues to be a grave concern in South Asia. Unless swift and targeted action is taken, some, if not all, of the countries of this region will fail to achieve the SDG by 2030.

## NUTRITION CHALLENGES IN SOUTH ASIA

The nutrition situation is particularly alarming in South Asia. Despite economic growth and a reduction in poverty, malnutrition is still rampant, and the region is the epicentre of a global stunting crisis [1]; researchers and policymakers term this situation the “South Asian Enigma”. It presents a paradox: on one hand, it is among the fastest growing developing regions [2], on the other hand, it also has the largest malnutrition burden. South Asia currently has the largest number of malnourished children in the world. A staggering 33.3% of all moderately or severely stunted children under five years, 15.3% of all moderately or severely wasted children under five years, and 3.1% of all overweight children under five years live in South Asia [3].

Looking at the levels and trends of the above mentioned five WHA nutrition indicators, it can be seen that (**Table 1**) although there has been remarkable progress on some indicators, this region is far from achieving the targets sets under the SDGs. The stunting rate has declined in all the countries in this region except Pakistan, with the lowest stunting rate observed in Sri Lanka. Between 2000 and 2015, there was a remarkable decline in Nepal (30.8 percentage points) and Bangladesh (29.7 percentage points); however, despite this progress, the rate remains high in Nepal (37.4%), Bangladesh (40.9%), Pakistan (45%), and India (38.4%). Between 2000 to 2015, though the rate of wasting fell in five countries, wasting rose in India (7.6 percentage points) and in Bangladesh (11.8 percentage points). While child malnutrition issues are a major concern in the region, they are eclipsed by the greatest cause of concern in the region: about 40%–50% of reproductive-age women in the region are anaemic.

**Table 1:** Trends in nutrition indicators in South Asia, 2000-2015*

	Stunting			Wasting			Anemia		
**Country/Year**	2000	2015	Absolute change	2000	2015	Absolute change	2000	2015	Absolute change
**Afghanistan**	53.2	40.9	-12.3	12.5	9.5	-3	36.6	42	5.4
**Bangladesh**	65.8	36.1	-29.7	2.5	14.3	11.8	48.1	39.9	-8.2
**Bhutan**	47.7	33.6	-14.1	17.1	5.9	-11.2	52.3	35.6	-16.7
**India**	57.7	38.4	-19.3	13.4	21	7.6	53.3	51.4	-1.9
**Maldives**	33	20.3	-12.7	11.3	10.2	-1.1	51	42.6	-8.4
**Nepal**	68.2	37.4	-30.8	14.2	11.3	-2.9	51.5	35.1	-16.4
**Pakistan**	42.7	45	2.3	15.5	10.5	-5	49	52.1	3.1
**Sri Lanka**	26.1	17.3	-8.8	17.5	15.1	-2.4	35.1	32.6	-2.5

*Source: Author’s calculations using UNICEF, WHO, and World Bank data (2019).

## 2030 PROJECTIONS FOR SELECTED COUNTRIES

**Table 2** presents projected levels for 2020, 2025 and 2030 for India, Nepal and Bangladesh, based on five-year compound annual growth rate (CAGR) between the year 2010 and 2015,. It can be seen that none of these countries are on track to achieve the SDGs by 2030. India will miss all the malnutrition targets by at least 20 percentage points, the anaemia target by 42.70 percentage points, and the exclusive breastfeeding indicator by 26.16 percentage points. Nepal has performed much better than India on most indicators: the rates of stunting are high, but Nepal has been successful in reducing wasting; it will miss the target by only four percentage points. Anaemia in women continues to be a cause of concern, just like in India. The problem of overweight children in Nepal is alarming; the projection shows that, by 2030, 17% of children in Nepal will be overweight (as compared with only 3% in India and Bangladesh). Bangladesh seems to be performing the best overall. It is on target to achieve the goal of exclusive breastfeeding, but anaemia among women continues to be high.

**Table 2:** Projections for business as usual and SDG target, prevalence rate in percentage*

	**BAU projections**	**SDG target**	

Country/Indicator	2015	2020		2025	2030	2020	2025	2030	Shortfall at 2030 (SDG target-projected)
**India**									
Stunting	38.40		33.96	30.04	26.57	20.26	10.69	5.64	20.92
Wasting	21.00		21.58	22.17	22.78	11.73	6.55	3.66	19.12
Anemia among women	51.20		50.12	49.07	48.03	24.09	11.33	5.33	42.70
Overweight	2.10		2.20	2.35					
Exclusive breastfeeding	54.87		60.26	66.17	72.67	66.76	81.22	98.82	-26.16
**Nepal**									
Stunting	37.40	32.08		27.51	23.60	19.74	10.42	5.50	18.10
Wasting	11.30	10.59		9.92	9.30	8.74	6.77	5.24	4.07
Anemia among women	34.90	31.28		28.04	25.13	1.62	1.26	0.97	24.16
Overweight	2.10	4.21		8.45	16.94				
Exclusive breastfeeding	56.68	58.69		60.77	62.92	67.32	79.95	94.96	-32.04
**Bangladesh**									
Stunting	36.10	31.59		27.64	24.19	19.05	10.05	5.31	18.89
Wasting	14.30	15.91		17.70	19.70	10.49	7.70	5.65	14.05
Anemia among women	39.70	37.08		34.63	32.34	20.95	11.06	5.83	26.50
Overweight	1.40	1.69		2.03	2.45				
Exclusive breastfeeding	55.29	69.96		88.52	100.0	69.93	88.44	111.8	On target

BAU – business-as-usual, SDG – sustainable development goals

*Source: Author’s calculations

**Figure:**
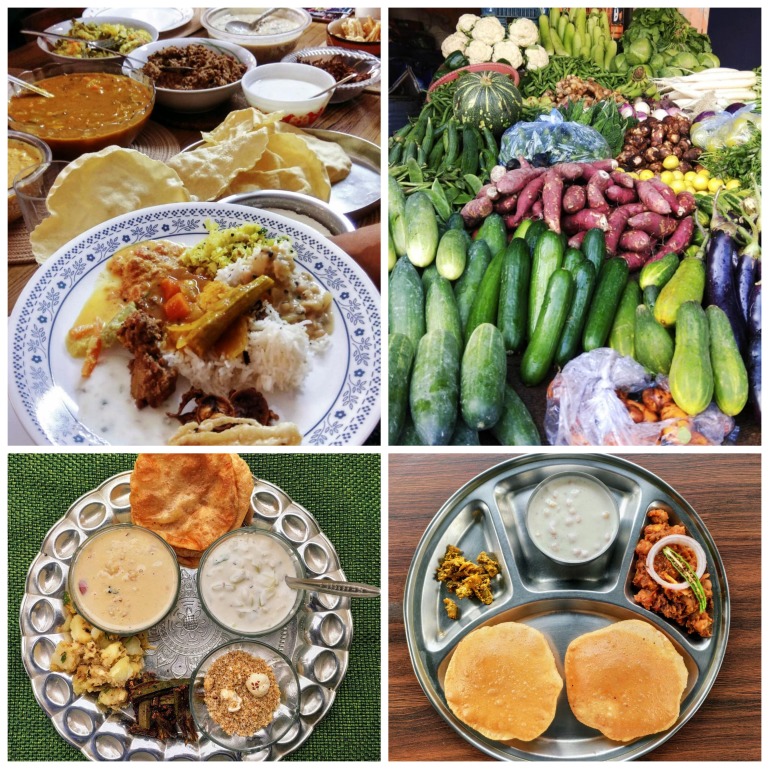
Photo: Local food and markets are good source of nutrition and well-balanced food in India (from the collection of Sonal Sardesai Gautam@ https://instagram.com/teaspoonrani, used with permission).

## A GROWING MENACE: ANAEMIA IN SOUTH ASIA

The projections of anaemia for South Asian women are alarming. By 2030, 48%, 25%, and 32% of women in India, Nepal, and Bangladesh, respectively, are projected to be anaemic. This is much higher than the target set by the WHA as well as the SDG target.

Anaemia in women, especially during pregnancy, contributes to poor birth outcomes, including prematurity and low birth weight [4]. It has also been suggested that about 23% of maternal deaths in Asia can be attributed to anaemia [5]. The consequences of anaemia are grave, not only for the current health status of women but also for the children being born to anaemic women, adding to the already high child malnutrition levels. The high levels of malnutrition among women can be ascribed to their secondary status and discrimination in South Asian society. Sen [6] points to the close link between women’s rights and health when referring to how social practices have led to the “missing millions” of women in South Asia due to higher female mortality. Women in South Asia have poor education, decision-making powers, and control over resources – all factors that limit their capability to acquire adequate nutrition. The causes of anaemia in the region vary by country, but they are largely linked to poverty, access to resources, and education. In India and Bangladesh, anaemia in women was found to be associated with lower wealth and lower education [6,7]. In Nepal, access to improved sanitation and drinking water facilities is associated with a lower rate of anaemia [8]. Other factors associated with high anaemia rates are diet diversity, education, and literacy [9]. It is therefore imperative to invest in women’s empowerment to improve the nutritional situation in the region.

## URGENT NEED TO ADDRESS WOMEN’S HEALTH

None of the South Asian countries appear to be on track to meet the SDG goal regarding malnutrition. In eradicating malnutrition, economic growth is the key, but only if it raises public development expenditure and reduces inequalities. A multispectral approach, not just a single factorial approach, is needed to reduce stunting; improvements in household asset accumulation, women’s education, and sanitation will improve nutrition. Given the importance of the region in terms of the number of malnourished people, it is crucial that the region meets the SDGs to improve the nutritional status of the global population. This study reiterates the well-established fact that the role of women will need to be strengthened within the region if the intergenerational cycle of malnutrition is to be broken.
